# Seeing beyond variables: applying a person-centered approach to identifying regulation strategy profiles among Finnish preclinical medical and dental students

**Published:** 2019-03-13

**Authors:** Henna Vilppu, Eero Laakkonen, Mirjamaija Mikkilä-Erdmann, Pekka Kääpä

**Affiliations:** 1 Department of Teacher Education and Centre for Learning Research, University of Turku, Finland; 2 Department of Biomedicine and Medical Education Research and Development Centre, University of Turku, Finland

## Abstract

**Background:**

High-quality learning during medical school and beyond requires appropriate study strategies and taking responsibility for one’s studies, thus self-regulation of one’s learning. In contrast to traditional studies focusing on a variable-centered approach, a person-centered approach to regulation strategies was utilized.

**Methods:**

The participants were 162 Finnish medical and dental students who answered the regulation scale of the Inventory of Learning Styles at three measurement points. First, the functionality of the scale was analyzed in Finnish medical education context. Latent profile analyses were used to examine regulation strategy profiles. Last, the connections of these profiles with the study success were investigated.

**Results:**

The analyses yielded a three-factor solution, which was reliable across time. Four profiles of regulation strategies were identified and they were found to be connected to study success: Students with the lowest self-regulation and increasing lack of regulation performed worse than the other groups.

**Conclusion:**

The use of a person-centered approach along with variable-centered approach increases understanding of the complex nature of learning in higher education. Person-centered approach could be used as a tool for supporting student learning and to help early diagnosing of learning difficulties, since it enables individualization of students with different regulation strategy profiles.

## Introduction

Medical schools aim to graduate doctors who are able to self-regulate their learning since this quality is required in the successful exercise of the medical profession.^1^^-^^3^ As the medical world is rapidly changing and renewing itself, it is important for medical doctors to constantly update their knowledge and skills, observe gaps in their knowledge, and search and evaluate new information.^4^ To benefit from continuous medical education, medical doctors have to be able to define their own learning needs, set goals for themselves, and engage in the most appropriate learning activities.^2^^,^^3^^,^^5^^,6^ Strong self-regulation skills also benefit students during medical school, as they confront the challenges of huge information load, time pressures, and accompanying stress.^7^ Furthermore, there is extensive evidence of the association between self-regulation and academic performance.^8^

In our study, we utilized the Inventory of Learning Styles (ILS)^9^ to investigate regulation strategies among medical and dental students during their first three years in medical school. At first, we examined the function of the regulation scales in the Finnish medical education context, and then we explored how medical and dental students regulate their learning across time and how this regulation is connected with study success. Our aim was to identify groups of students with different regulatory profiles by using a person-centered approach to the investigation of regulation strategies. The main idea behind person-centered approach is that an individual’s score on a single dimension derives its meaning from the scores the same individual has on other dimensions (as opposed the scores other individuals have on the same dimension, as in variable-centered approach).^10^ Further, the person-centered approach aims to form homogenous subgroups of people with similar profiles across variables and then relate these profiles to an external variable, such as study success in our study (as opposed to simple correlations between different variables in variable-centred approach).^11^

### Self-regulated learning vs. three qualitatively different regulation strategies of learning

Over the past decades of research, multiple models and operationalisations of self-regulated learning (SRL) have been presented, and there is still no universally accepted definition of SRL.^12^ However, consensus seems to exist among researchers that SRL is the ability to actively monitor and regulate one’s learning by using various cognitive, metacognitive, and behavioural strategies, such as exerting effort and self-testing.^13^^-^^16^ When the learning outcomes are considered, researchers have found and reported that self-regulated learners often seem to be the most successful and effective learners.^17^^-^^22^

Related to the multiple conceptualizations of SRL, Winne and Perry^15^ differentiate between models that measure SRL as an event (process models) and the ones that measure SRL as an aptitude (component-oriented models).^12^ The former measure SRL as consecutive regulatory phases of situation-specific learning processes, whereas the latter models are more general, asking the respondents to abstract over multiple SRL events and generalize their actions across situations. Further, the latter models include multiple components of SRL, such as metacognitive and cognitive strategies.^12^ The ILS questionnaire used in this study represents a component-oriented approach and it can be used to assess students’ competencies regarded as prerequisites of SRL without taking the actual SRL process into account. Vermunt and colleagues^21,^^23^^-^^26^ define self-regulation of learning as metacognitive activities, which include planning, monitoring, testing, and evaluating one’s learning processes. Thus, their definition is not as comprehensive as the ones including affective and behavioural aspects of student-regulated learning processes.^14^^,^^16^ However, what is unique in their theory is that they distinguish self-regulation of learning from other qualitatively different types of regulation of learning, i.e. from external regulation and lack of regulation.^23^^,^^24^ External regulation is the regulation of learning by teachers, study materials, or other aspects of the learning environment. In this situation, the responsibility for learning is given to the teacher, who plans, sets goals, evaluates, etc. Students who experience lack of regulation notice that they have problems in learning, but do not know how to do it differently and better. Further, they have difficulty evaluating whether they have mastered certain content. Typically, these students strongly direct themselves toward the regulation supplied by the instruction, but they find it inadequate to support their learning.^23^

In higher education settings, such as medical school, self-regulation seems to be the most appropriate strategy because external support is very limited.^26^ University studying requires students to be proactive and self-disciplined learners capable of controlling their learning via self-monitoring and self-evaluation.^8^ Additionally, self-regulatory skills are important after graduation, since many skills in work life are learned informally, without much external support. One of the main purposes of all higher education is to help students develop lifelong learning skills, in which self-regulation plays an important role.^17^^,^^21^^,^^22^^,^^27^

### A short overview of the structure and previous studies of the ILS

The ILS was developed in a European higher education setting for researching students’ learning patterns.^23^^,^^24^ In addition to regulation strategies, the complete ILS covers cognitive processing strategies, mental models of learning, and learning orientations. The conception of learning underlying the ILS is that mental models of learning and learning orientations affect regulation of learning, which in turn influences processing strategies.^24^ Combinations of these learning dimensions form four qualitatively different learning patterns: undirected, reproduction-directed, meaning-directed, and application-directed.

Characteristic to an undirected learning pattern is that students hardly process the subject matter since they have trouble with selecting what is important within huge amounts of material to be learned.^28^ Further, these students show lack of regulation in their studying as the value of the learning is provided by other students and teachers, and they have an ambivalent learning orientation (expressing doubts about their study choices and their own capacities, for instance). The second learning pattern, reproduction-directed way of learning, is characterized by a stepwise processing strategy (e.g., memorizing, rehearsing), external regulation, view of learning as the intake of knowledge, and certificate and self-test oriented learning orientation. Students with the third learning pattern, meaning-directed, make use of a deep processing strategy (e.g., relating, critical processing), self-regulate their learning, see learning as a personal construction of knowledge and have a personal interest in the subject matter as their learning orientation. Students who manifest the fourth learning pattern, application-directed, use a concrete processing strategy (e.g., concretize the subject matter), involve both self and external regulation strategies, view learning as being able to use the knowledge they have acquired, and are vocation oriented in their learning motivation.^28^

Learning patterns represent a temporal interplay between personal and contextual influences rather than an unchangeable personality attribute.^23^ Previous research has found evidence for changes in learning patterns during higher education.^29^ Students seem mainly to develop toward a more meaning-oriented or application-oriented learning pattern and move away from an undirected learning pattern.^30^ Also, development within a learning pattern from external to internal regulation seems to exist.^23^ Thus, the more experienced and skilled students become in a certain learning pattern the more they execute it under internal control. Students adopt a certain pattern until they experience friction between their current learning pattern and the demands of the learning environment, for example, higher education, or for some other reason become dissatisfied with their approach to learning. Then an alternative pattern is adopted, first under external control. As the learning-pattern model^24^ suggests, processing and regulation strategies seem to be more prone to change than mental models of learning or learning orientations.^29^^-^^31^

The complete ILS or some parts of the inventory have previously been used with medical students at least in Belgium,^32^^,^^33^ Finland,^34^^,^^35^ Norway,^5^^,^^36^ Sri Lanka,^37^^,^^38^ Sweden,^39^ Turkey,^40^ and Vietnam.^41^ However, the results of these studies are to some extent inconsistent, and making comparisons between them is complicated since several adaptations have been made to the original inventory. According to Finnish studies, novice medical students often express external regulation and reproduction-directed learning, whereas advanced medical students seem to be more application-directed.^34^^,^^35^ Other studies focused mainly on curricular issues. They have shown, for instance, that curriculum innovations may^32,^^33^^,^^37^ or may not^36^ affect regulation strategies, and that a significant association between academic performance and frequent use of deep processing strategies or self-regulation does not necessarily exist.^38^ However, according to Lindblom-Ylänne and Lonka,^34^ meaning-oriented independent medical students performed better during preclinical studies than reproduction-oriented and externally regulated students. Further, only a few studies were longitudinal, and their focus was on the impact of curriculum changes or different curricula on learning patterns or some aspects of them.^5^^,^^32^^,36^ Thus, there seems to be a lack of follow-up studies focusing on the qualitative differences in medical students’ regulation of learning across time.

### The study

In our study we aimed at identifying subgroups of medical and dental students who show different profiles of regulation strategies during the first three years of medical school. Additionally, we examined the connections between these profiles and study success. We used latent profile analyses (LPAs) to explore a person-centered approach, i.e., to identify groups of students with similar profiles of regulation strategies and validated these groups against a study success measure. Traditionally, the approach to studying learning patterns has been variable-centered, meaning that the structure of learning pattern factors has been examined across individuals or the special effect of some learning pattern factors or the relation of each learning pattern factor to other variables has been examined.^20^^,^^30^^,^^31^^,^^42^ Similarly, the impact of self-regulation on student learning has usually been studied by variable-centred analytical approaches focusing on the relationships between self-regulated learning variables and student outcomes.^8^ Therefore, in a sense, we wanted to see the students behind these variables by choosing the more idiographic, person-centered approach.^43^

In the present study, we followed the same group of students during their first three years at medical school. At first, the function of the regulation scales was examined in a medical education context as a preparatory analysis for answering the research questions (Part 1). The research questions of the study were as follows: a) What kind of regulation strategy profiles can be identified among medical students across time (Part 2)?; and b) To what extent do regulation strategy profiles of students predict study success in preclinical studies (Part 3)?

## Methods

### Context, participants, and procedure

The participants of the study were 162 native Finnish-speaking students from a Finnish medical faculty with a traditional medical curriculum. The six-year medical education programme is divided into preclinical and clinical phases. Two first academic years of the curriculum are preclinical, meaning that students focus on general and basic sciences. The first semester of the third academic year consists of the basics of clinical medicine, whereas from the spring semester onwards, the students move on to clinics. Half of the students (*n* = 89, 55%) were women, and the rest were men (*n* = 73, 45%). A total of 123 students were studying medicine (61 male, 62 female), while 39 were studying dentistry (12 male, 27 female). All participants had gone through a demanding selection process (in Finland, only approximately 15% of applicants are accepted into medical school). The students answered a shortened version of the regulation strategy scale of the ILS (original version)^9^ three times during their first three years in medical education, thus, in 2009 (*N* = 162), 2010 (*N* = 118), and 2011 (*N* = 110). The number of participants decreased slightly every year, but the comparisons between drop-outs and the remaining participants indicated that these groups did not differ significantly (*p*>.05) from each other in terms of regulation strategies, sex, or training program (medicine vs. dentistry). Participation in the study was voluntary and written informed consent was obtained from the participants. The study was approved by the authors’ Research Ethics Board.

### Materials

In the original version of ILS,^9^ 28 items in total measure regulation strategies: 11 self-regulation items (including subscales of self-regulation of learning processes and results and self-regulation of learning content), 11 external regulation items (including subscales of external regulation of learning processes and external regulation of learning results), and six lack of regulation items.

We made some minor changes to the wording of the original scales based on the feedback given by medical students and teachers in face-to-face interviews. Further, we omitted some of the items from the original scale because they were considered to fit poorly in the context of Finnish medical education. In particular, the items measuring external regulation of learning results seemed to poorly describe the learning context of this study, since, for example, there usually are no assignments or tasks in the textbooks. Further, since the teaching in the preclinical phase in a traditional medical curriculum is mostly based on lectures, the teachers do not give many assignments or exercises. Thus, the version we used included 20 items in total: 10 items concerning self-regulation, four items concerning external regulation, and six items concerning lack of regulation. Shorter versions of the ILS have also been used in other studies.^44^^,^^45^ We asked students to indicate on a 5-point scale (1 = *completely disagree*, …, 5 = *completely agree*) the degree to which the phrased activity corresponded with their own studying.^cf.24^ The purpose of the changes we made in the original scales was to improve the cultural appropriateness of the instrument and make it relevant for Finnish higher education. Examples of the items used are: “*When I start reading a new chapter or article, I first think about the best way to study it*” (self-regulation of the learning processes and results), and “*I have trouble processing a large amount of the subject matter*” (lack of regulation).^24^

Study success was measured with general performance assessment (GPA) score in preclinical studies. Before entering to supervised clinical practice, medical students within a certain year are ranked based on the grades they have achieved during the previous semester. The course credits of each course are multiplied with the course grade of each course, and then all these scores are added up to form a GPA score in preclinical studies for each student. Thus, we considered GPA score a comprehensive indicator of study success in preclinical studies.

### Data analysis

#### Part 1: Preparatory analysis of the measures - Testing the regulation scales in medical education

We considered testing the ILS regulation scales in the context of Finnish medical education necessary because of the changes made to the original scales (see the Materials section). To explore the measurement model of the regulation scales, we conducted statistical analyses of the second measurement data to examine the underlying factor structure. We chose the second measurement point because after the 1st year the students would have adapted to medical school, and learning strategies used during previous studies, like high school, would no longer dominate. Further, during the 3rd year, students move on from the preclinical phase to the clinical phase, which might again affect their regulation of learning. Thus, we considered the 2nd study year the best one for confirming the factors.

We calculated descriptive statistics and correlations for each item at the second measurement. Then we performed a confirmatory factor analysis (CFA) to test the theory-based factor structure. We estimated the CFA models by using full information maximum likelihood estimation with robust standard errors (MLR), which can handle missing and non-normal data. Model fit was evaluated using the chi-square test and fit indices with general cutoffs:^46^^,^^47^ the chi-square test (nonsignificant *p* value indicates good fit), the comparative fit index (CFI) and the Tucker-Lewis index (TLI; values 0.90 or above indicate acceptable fit), the root mean square error of approximation (RMSEA), and the standardized root mean square residual (SRMR; values 0.08 or below indicate acceptable fit). To test the factor structure in each time point, we evaluated the model fit of the CFA models across time. Longitudinal CFA-models with measurement invariance analyses were impracticable due to the small sample size. Further, we compiled the composite scores based on the CFA-model, and calculated descriptive statistics and Cronbach coefficients for the regulation subscales at each measurement. In addition, we computed correlations over time for the regulation strategies to further test the reliability of the scale.

#### Part 2: Identification of student subgroups

To identify subgroups of students who showed differences in regulation strategies of learning, we used a clustering approach. This was done by using a mixture analysis as implemented in the Mplus programme.^48^ Mixture modelling is based on the idea that the observed data can represent subpopulations, i.e., latent classes, and that these classes can be identified. Compared to the traditional cluster analysis, the advantage of the mixture model analysis is that it uses statistical criteria to decide the number of latent classes. Of the many model variations the Mplus program provides, we chose LPA (see e.g.,^49^) in order to identify the smallest number of latent groups that describe the variance of regulation scales.

We carried out LPA for the three measurement results (T1, T2, and T3) using the students’ composite scores of regulation strategies (self-regulation and lack of regulation). We fitted the models with different numbers of latent subgroups, and compared these model solutions using the model fit information in order to determine the best solution. To evaluate the appropriate number of latent subgroups, we used three criteria: a) the fit of the model; b) the distinguishability of the latent groups evaluated according to entropy (entropy values range from 0 to 1, where values close to 1 indicate a clear classification) and the average latent group posterior probabilities; and c) the usability and interpretability of the latent subgroups in practice. Model fit was evaluated using the log-likelihood values (log L), Akaike’s information criterion (AIC), the Bayesian information criterion (BIC), and the Vuong-Lo-Mendel-Rubin test (VLMR). Higher log-likelihood value indicates a better fit. Lower AIC and BIC values indicate a better model, and significant VLMR test results indicate a higher number of latent subgroups. We estimated all models using the MLR estimation method.

#### Part 3: Connections between regulation strategies and study success

In the third part of the study, we examined the connections between regulation strategies and study success. We compared the student groups created in LPA in relation to study success, which was measured with the GPA score in the preclinical studies. Analyses were performed using the BCH method for 3-step mixture modelling with continuous distal outcomes.^50^^,51^ In BCH-procedure, an overall test with multiple comparisons is made for class-differences so that the posterior probabilities for class membership are taken into account.

## Results

### Part 1: Preparatory analysis of the measures - Testing the ILS regulation scales in medical education

** To validate the shortened version of ILS regulation scales in medical education, we calculated descriptive statistics and correlations for the 16 items measuring different aspects of regulation of learning (see Table A1 in Appendix A). Due to weak inter-item correlations and low internal consistency (α < .60), we omitted the four items concerning external regulation from further analyses. As can be seen in the Appendix A, the most powerful correlations exist between items SR4–SR11 (.14–.66), SR2–SR9 (.39–.60), and LR1–LR6 (.18–.55). This supports the theory behind the inventory, i.e., that there are different regulation strategies, lack of regulation (LR1–LR6), and two separate dimensions of self-regulation: self-regulation of learning processes and results (SR4–SR11) and self-regulation of learning content (SR2–SR9) (see the items in Appendix B). Thus, verifying the regulation strategy scales behind the items seemed reasonable.

**Figure 1:**
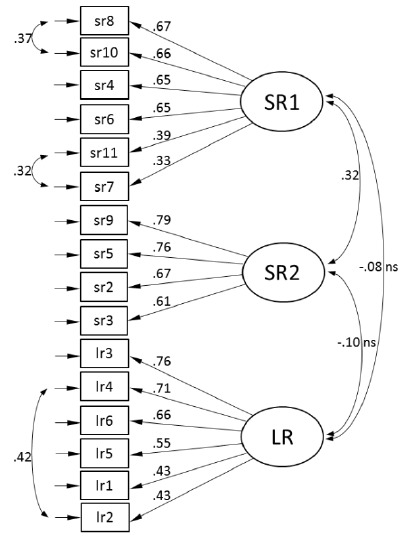
CFA model at time 2 (standardized solution)

Note. Model-fit information: *χ^2^*(98) = 113.96, *p* = .13, RMSEA=.04 (95% CI: .00-.06), CFI= .96, TLI= .95, SRMR= .08. SR1 = self-regulation of learning processes and results, SR2 = self-regulation of learning content, LR = lack of regulation.

Next, we analyzed the theory-based structure of the three factors with CFA (Figure 1). CFA with three factors resulted in a coherent model with no cross-loadings. However, the model required three residual covariances (between SR11 and SR7, SR8 and SR10, and between LR4 and LR2). The covariance between the items seemed justified, since the items shared the same idea or very similar wording. The fit of the final model was acceptable.

To test the stability of the factor structure across time, we calculated the model fit of the CFA models across the three measurement points (Table 1). According to the results, the three-factor model illustrated in Figure 1 seemed to fit the data reasonably in the first and third measurements (acceptable to mediocre fit). This result indicates the structural validity of the model. However, the model fit was the best at the second measurement (highest CFI and TLI, lowest chi-square, RMSEA and SRMR).

**Table 1:** Model fit of the CFA models across time

Time	*χ^2^*(*df*)*, p*	CFI, TLI	RMSEA	SRMR	AIC, BIC
Time1 (*N*=162)	167.88 (98), .000	.89, .86	.06	.09	6386.51, 6553.24
Time2 (*N*=118)	113.96 (98), .129	.96, .95	.04	.08	4922.35, 5071.97
Time3 (*N*=110)	136,78 (98), .006	.90, .88	.06	.08	4915.83, 5061.66

*Note*. χ^2^ (*df*) = chi-square test of model fit with degrees of freedom and p-value, CFI = comparative fit index, TLI = Tucker-Lewis fit index, RMSEA = root mean square error of approximation, SRMR = standardized root mean square residual, AIC, BIC = information criteria.

Next, we formed sum scales of the regulation strategies based on the CFA model, and their reliabilities were examined in each wave (Table 2). The levels of different regulation strategies seem to be consistent throughout the different measurement points. For example, the level of self-regulation of learning processes and results (SR1) seems to remain lower than the level of self-regulation of learning contents (SR2) across time. The scales were fairly normally distributed (skewness and kurtosis between ±1 and non-significant), and the reliabilities estimated with the Cronbach α coefficient seemed acceptable (>.60-.70).^52^

**Table 2:** Descriptive statistics and Cronbach coefficients of the regulation scales

	*N*	*Min*	*Max*	*Mean*	*SD*	*SK*	*Rku*	*Alpha*
**Time1**								
SR1	150	1.00	4.67	2.74	.76	.03	–.42	.83
SR2	146	1.25	5.00	3.06	.70	.27	.18	.70
LR	152	1.17	4.83	2.83	.64	.26	.46	.74
**Time2**								
SR1	108	1.00	4.17	2.73	.72	–.08	–.45	.61
SR2	109	1.00	5.00	2.97	.85	.11	–.34	.80
LR	108	1.33	4.67	2.80	.71	.34	.05	.79
**Time3**								
SR1	110	1.00	4.50	2.85	.79	–.06	–.78	.81
SR2	110	1.00	5.00	3.32	.66	–.16	.71	.62
LR	110	1.00	4.83	2.95	.72	–.06	–.31	.72

*Note*. SR1 = self-regulation of learning processes and results, SR2 = self-regulation of the learning content, LR = lack of regulation.

Next, we examined the interrelations and stability of the regulation scales (Table 3). The correlation analysis over time supported the reliability of the measures, since the SR1 scale correlated strongest with the SR1 scales of the other time points, the SR2 scale with other SR2 scales of the other time points, and the LR scale with the other LR scales.

**Table 3:** Correlations of the regulation scales

	SR1_T1	SR1_T2	SR1_T3	SR2_T1	SR2_T2	SR2_T3	LR_T1	LR_T2	LR_T3
*Self-Regulation1*									
SR1 Time1	1.00								
SR1 Time2	.61	1.00							
SR1 Time3	.59	.75	1.00						
***Self-Regulation2***									
SR2 Time1	.34	.05	.01	1.00					
SR2 Time2	.43	.23	.32	.52	1.00				
SR2 Time3	.31	.19	.31	.51	.65	1.00			
***Lack of Regulation***									
LR Time1	.09	.03	.07	.07	–.10	–.01	1.00		
LR Time2	.09	.06	.07	.09	–.06	–.12	.54	1.00	
LR Time3	–.16	–.19	–.20	–.13	–.27	–.21	.42	.60	1.00

*Note*. If |*r*| > 0.14 or |*r*| > 0.19 or |*r*| > 0.26 then correlation is significant at the level of *p* < .05, *p* < .01, *p* < .001, respectively.

To conclude, we considered a three-factor model that included two subscales of self-regulation and one scale concerning lack of regulation to fit well in examining Finnish medical students. Further, the reliability of the scales was verified across time.

### Part 2: Identification of student subgroups

The second aim of the study was to investigate what kind of regulation strategy profile groups could be found among the students. We formed sum scales in each of the three measurement points on the basis of the CFA three-factor solutions and used these in LPAs. Because of the small sample size, we chose only one self-regulation factor (SR1, self-regulation of learning processes and results) and a lack of regulation factor (LR) to the LPAs to reduce the large number of the estimated parameters in the model. We chose SR1 because it reflects self-regulation of learning processes and results, i.e., planning, monitoring and evaluating, and gives thus a more general view of self-regulation than SR2 that specifically refers to self-regulation of learning contents. Table 4 presents the fit indices and class sizes for the solutions with different numbers of latent profile groups.

**Table 4:** Fit indices from latent profile analysis (LPA) with different number of latent profile groups

Numberof groups	BIC	Entropy	VLMR (*p*)	Size of group *n*	Group assignment probabilities for each group	Number of estimated parameters
1	2118.35			159	1.00	12
2	2078.06	.69	.007	95, 64	.90, .91	19
3	2064.59	.70	.723	43, 68, 48	.86, .82, .92	26
4	2057.45	.76	.053	49, 55, 48, 7	.81, .94, .84, .97	33
5	2063.10	.74	.234	41, 40, 28, 45, 5	.78, .77, .86, .92, .97	40
6	2072.19	.74	.323	26, 41, 27, 38, 22, 5	.86, .78, .87, .78, .83, .95	47

*Note*: BIC = Bayesian information criterion, VLMR = Vuong-Lo-Mendel-Rubin test *p* value.

We chose the four-group solution for further analyses based on the fit of the models, the distinguishability of the latent groups, and theoretical considerations. Of the three indices, the BIC and entropy suggested the four-group solution: The BIC value increased when the five group-solution was used, and the entropy value was highest for the four-group solution. The VLRM test suggested a two-group solution, but the test result was almost statistically significant also in the four-group solution. According to Nylund, Asparouhov, and Muthén^53^, the BIC has been shown to be the most reliable index; hence it was chosen as a criterion. Further, in five and six cluster solutions group assignment probabilities for some groups would have been below .80.^54^ Finally, the four-group solution’s interpretability was best on theoretical grounds providing a logical combination of learning strategies.

**Figure 2:**
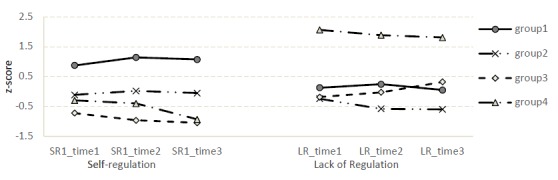
The profiles of self-regulation and lack of regulation for four latent groups

Note. Group 1: High SR-Average LR (*n* = 49), Group 2: Average SR-Low LR (*n* = 55), Group 3: Low SR-Average LR (*n* = 48), Group 4: Low SR-High LR (*n* = 7).

Figure 2 presents the standardized means of the final LPA solution with four regulation profiles. The first profile group showed high self-regulation and average lack of regulation (*n* = 49, 31%), and will be referred to as the High SR-Average LR group. The second latent profile group was characterized by an average level of self-regulation and low lack of regulation (n = 55, 35%), and is thus called the Average SR-Low LR group. The third latent profile group showed low levels of self-regulation and average levels of lack of regulation. The group was comprised of 48 (30%) students and will be referred to as the Low SR-Average LR group. The last latent profile group demonstrated low self-regulation and high lack of regulation. There were seven (4%) students in this group that is referred to as the Low SR-High LR group.

Within the four groups, self-regulation of the learning processes and results as well as lack of regulation appeared to be rather constant, although minor changes can be seen (see Figure 2). In groups 1 and 2, self-regulation slightly increased from first year to the second year, but after that it remained quite stable. In groups 3 and 4, self-regulation decreased from the first study year to the third. Lack of regulation decreased towards the third study year in all groups except group 3, where it increased. Compared to other groups, group 1 had clearly the highest scores on self-regulation, whereas the group 4 had an extremely high lack of regulation scores.

#### Part 3: Connections between regulation strategy profiles and study success

Last, we examined the connections between regulation strategy profiles and study success. Study success was measured with GPA score in preclinical studies (*N* = 101, *M* = 266.05, *SD* = 67.81, *Min* = 93.00, *Max* = 437.00). We analyzed the data using LPA-model with GPA as a distal outcome.

Group 1 (High SR-Average LR, *n* = 49) had the highest GPA score (*M* = 285.22, *SD* = 82.54), group 2 (Average SR-Low LR, *n* = 55) the second highest (*M* = 284.49, *SD* = 103.40), and group 3 (Low SR-Average LR, *n* = 46) had the lowest GPA score (*M* = 230.91, *SD* = 93.04). Group 4 (Low SR-Average LR, *n* = 7) also had low GPA-level (M = 241.63, SD = 92.11). The differences in mean levels between the groups were significant (*χ*^2^(3) = 10.77, *p* = .013). Pairwise comparisons showed that the Low SR-Average LR Group performed statistically significantly worse in their studies than the groups 1 and 2 (*χ*^2^(1) = 9.11, *p* = .003 and *χ*^2^(1) = 5.94, *p* = .015, respectively). The other group-differences concerning study success were not significant. In sum, we were able to identify students with differing regulation profiles, which were connected to study success.

## Discussion

The current study extends the existing literature by adopting a person-centred approach to investigate the differences in medical and dental preclinical students’ regulation strategies of learning during the first 3 years in medical school. At first, the function of the regulation scales of the ILS was examined as a preliminary analysis. Finally, the connection between regulation strategy profiles and study success was explored.

Our study provides general evidence that the regulation strategy scale of the ILS with the exception of external regulation is a valid construct within the context of Finnish medical education. Analyses resulted in a three-factor model, which includes three scales of regulation of learning: lack of regulation and two subscales of self-regulation, self-regulation of learning processes and results, and self-regulation of learning content. We had to exclude the external regulation scale from the analyses because of poor reliability. Problems with the scale have also emerged in other studies.^28^^,^^32^^,^^39^^,^^55^ The three-factor solution was repeatable and thus reliable across the first three years in medical school. The model fit of the three-factor model was best at the second measurement, as we expected. We argue that by the 2^nd^ study year the students had adapted to the lecture-based preclinical phase of medical school, and the high-school regulation strategies would no longer dominate.^30^ During the 3^rd^ study year the learning environment changes to a more practical clinical phase, which might again affect learning strategies.

To accomplish the second aim, we used LPAs to identify different groups in terms of regulation strategies. We found four distinctive regulation profiles: High SR-Average LR (group 1), Average SR-Low LR (group 2), Low SR-Average LR (group 3), and Low SR-High LR (group 4). The students divided quite evenly between the three first profiles, but the representation of the fourth profile was considerably smaller. The overall results of different combinations of self-regulation and lack of regulation resonate with the findings of Donche and Van Petegem^56^ who state that the ways in which most students engage in learning can seldom be characterized by a single learning strategy. Rather, students combine several regulation strategies, to a different degree, thus forming relatively unique regulation profiles. Throughout the three-year measurement period, the levels of self-regulation and lack of regulation remained rather stable within the groups. This result came as a surprise, given the support from several other research findings for changes in learning patterns^29^, and especially in processing and regulation strategies during higher education.^29^^-^^31^ On the other hand, Strømsø et al.^36^ reported lack of change in study strategies and suggest that more general study strategies included in the questionnaire were not affected in a more vocational learning environment, such as medical school. Also, in a study by Lucieer et al.,^2^ the levels of most self-regulated learning skills did not differ between first- and third-year medical students, indicating that these might not develop much during medical school. Reasons for the stability of self-regulation skills could be that the medical curriculum is highly structured, and thus does not leave much space to students to develop these skills.^3^ Possibly, as only the best students are accepted to medical school, they might already possess relatively high regulation skills, and thus show little development of these skills during their studies (i.e., ceiling effect).^2^ Further, Lucieer et al.^2^ argue that although the curriculum would emphasize self-regulated learning skills, it might be that medical schools too early assume that students develop these skills themselves, without explicitly teaching them to do so.

Lastly, we investigated the connections between the regulation strategy profiles and study success. The student group who showed increasing lack of regulation and lowest self-regulation (group 3) had a poorer GPA score than the others. The results are in line with previous studies, which indicate that lack of regulation and other aspects of undirected learning style are consistently and negatively associated with the students’ exam achievements^20,^^34^, and further, that self-regulation is connected to study success.^34^^,57^ One hypothesis for the association between good grades and self-regulation (or bad grades and lack of regulation) could be that they are both caused by prior learning. For example, students with a strong background in biomedical knowledge probably do not find the curriculum overly challenging, and therefore they have resources to engage in self-regulated learning. Whereas those self-regulated learners, who have not acquired such a strong biomedical knowledge background, might more easily lose control over their learning in the crowded curriculum. This might explain the finding that in a highly selective student population almost a third of the students demonstrated a maladaptive regulation pattern of increasing lack of regulation and lowest self-regulation. The similar results have been found in a study by Heikkilä et al.,^58^ in which a majority of students represented a non-regulating profile type, with highest levels of stress and lack of interest in academic studies. Also, in a sample of Chinese final-year university students, the largest profile type with third of the participants was termed minimal self-regulated students, indicating low levels of motivation, attitude, and academic self-concept.^8^ One possible explanation for a high representation of maladaptive regulation patterns might be that there is a mismatch between the students and their learning environment, that the learning environment does not offer enough support for the development of self-regulation skills.^58^ It might be that in the early stages of studies medical students are more dependent on external regulation and support than what is expected of them, likely because of the overcrowded curriculum. It seems obvious that students would need more support in developing their self-regulation of learning.

Data-gathering from the whole cohort, longitudinally over the first three years of medical school, contributes to the strength of the study. The use of a person-centred perspective on regulation strategies increases understanding of the complex nature of university students’ learning, and might help in translating complicated models into educational practice.^10^ Further, by identifying typical profiles within a student population and their relations to other variables, valuable and complementary information to variable-centred approach can be yielded.^10^ Although there are some limitations concerning the ILS, it seems to offer an adequate background variable for the needs of multi-methodological educational science. The study shows that even by using a shortened version of the ILS regulation scale within a highly selected sample it is possible to detect diverse regulation profiles that predict study success.

Some limitations of the current study should be considered. First, as the study is based on students’ self-reports, as are many other studies on regulation of learning, it does not reflect students’ actual learning behaviour, but their conceptions of their regulation.^30^ Thus, other assessment methods should be considered together with self-report measures. Further, as the ILS was originally developed for higher education learning in general,^24^ more domain-specific measurement of regulation strategies might be appropriate in the future in strongly vocationally oriented learning environments, such as medical school. Or, as suggested by Edelbring,^39^ one approach would be to add a vocationally oriented scale along with the other regulation scales of the ILS. It would also be interesting in the future to triangulate the ILS scores with one of the more situation specific and process-oriented models of SRL to see how these are connected.^12^

Despite these limitations, the current study expands our understanding of regulation strategies during the early years of medical school. The results of the study could be used to support curriculum design and medical educators, and help developing the teaching practices in the demanding medical curriculum. If we aim at educating self-regulated doctors, it is important to recognize that students’ learning strategies might be affected by the way educational programmes are organized.^5^^,^^6^ For example, by increasing active teaching methods, students’ skill in self-regulated learning could be enhanced. Further, by paying attention to the assessment practices, external regulation could perhaps be reduced. Thus, medical schools should evaluate their curriculum to see to what extent they stimulate the development of SRL skills and whether there are any factors, such as the overcrowded content in the curriculum, that may hinder this development.^2^ Promoting SRL in a few courses is not enough, instead, active incorporation of strategies to facilitate SRL is needed throughout the curriculum.^3^ Still, providing individual support and qualitative feedback for huge student masses remains a challenge.

Lack of regulation has been associated with problems in students’ well-being, such as relatively high levels of stress and exhaustion.^58^^,^^59^ It might be that students with high SRL skills are better able to cope with the stress caused by the crowded curriculum, since they know where to focus and what are the most efficient ways of studying the material, whereas students who experience lack of regulation are lost in their studies and thus more stressed. Alternatively, students with a strong background in biomedical knowledge have better resources for deepening their understanding in a self-regulated manner than those students with weaker biomedical prior learning and are more likely to experience fewer academic problems. The person-centred approach could be used as a tool for student counselling to support learning and help early diagnosing of learning difficulties in higher education, since it enables individualizing students with different regulation profiles. The possibility to recognize students at risk with the help of this tool could be important in order to offer timely and tailored support, such as reducing stress and helping them to manage with the overcrowded curriculum. In medical education, where the pace of studies is fast and the risk of being left behind or even dropping out is great, early intervention is especially important.

## Appendix A

**Table A1:** Descriptive statistics and correlations of the items (Time 2, N = 118)

	SR4	SR6	SR7	SR8	SR10	SR11	SR2	SR3	SR5	SR9	LR1	LR2	LR3	LR4	LR5	LR6
***Mean***	2.63	2.39	2.37	3.13	2.78	3.07	3.83	2.40	3.16	2.62	2.79	2.29	2.78	2.82	3.01	2.98
***SD***	1.05	1.12	1.04	1.04	1.12	1.02	.89	1.02	1.18	1.17	.89	1.05	1.01	1.07	1.04	1.07
***SK***	.15	.45	.40	–.51	.01	–.42	–.84	.35	–.19	.45	.65	.53	.14	.14	.04	.30
***Rku***	–1.14	–.91	–.99	–.45	–.86	–.55	.51	–.41	–.99	-.66	–.23	–.55	–.87	–.79	–.93	–.88
***Correlations*** **(*****r*****)**																
**SR4**	1.00															
**SR6**	.46	1.00														
**SR7**	.14	.23	1.00													
**SR8**	.43	.45	.25	1.00												
**SR10**	.42	.37	.27	.66	1.00											
**SR11**	.19	.18	.40	.36	.34	1.00										
**SR2**	.15	.26	.05	.02	.11	.08	1.00									
**SR3**	.08	.13	.08	.02	.20	.10	.45	1.00								
**SR5**	.21	.17	–.02	.09	.20	–.06	.54	.39	1.00							
**SR9**	.25	.26	.10	.11	.26	.13	.47	.56	.60	1.00						
**LR1**	–.13	–.10	–.07	–.15	–.17	.02	–.19	–.09	–.05	–.01	.00					
**LR2**	.00	.09	.25	.10	.08	.31	.04	.04	.05	.18	.23	1.00				
**LR3**	–.01	.10	.10	–.04	–.09	.10	–.04	–.12	–.05	.04	.38	.40	1.00			
**LR4**	–.10	.03	.19	–.08	–.12	.19	–.07	–.15	–.03	.00	.28	.56	.55	1.00		
**LR5**	–.04	–.03	.02	–.06	–.11	.04	.00	.02	.10	.18	.22	.18	.45	.43	1.00	
**LR6**	–.17	–.02	.17	–.04	–.20	.18	–.04	–.11	–.15	–.05	.20	.30	.55	.50	.29	1.00

*Note*. If |*r*| > 0.16 or |*r*| > 0.22 or |*r*| > 0.30 then correlation is significant at the level of *p* < .05, *p* < .01, *p* < .001, respectively. SR4–SR11 = self-regulation of learning processes and results, SR2–SR9 = self-regulation of learning contents, LR1–LR6 = lack of regulation.

## Appendix B

**The items included in the analyses (adapted from ILS^9,24^)**

**Self-regulation of learning processes and results**

SR4. To test whether I have mastered the subject matter, I try to think up other examples besides the ones given in the study material or by the teacher.

SR6. To test my learning progress, I try to answer questions about the subject matter which I make up myself.

SR7. When I start reading a new chapter or a complex of issues, I first think about the best way to study it.

SR8. To test my own learning, I try to describe the content of a chapter in my own words.

SR10. To test my learning when I have studied a text book, I try to formulate the main points in my own words.

SR11. When I have difficulty grasping a particular piece of subject matter, I try analyse why it is difficult for me.

**Self-regulation of learning contents**

SR2. If I do not understand a study text well, I try to find other literature about the subject concerned.

SR3. I do more than I am expected to do in a course.

SR5. I add something to the subject matter from other sources.

SR9. In addition to the syllabus, I study other literature related to the content of the course.

**Lack of regulation**

LR1. It is not clear to me what I have to learn and what do I not have to learn.

LR2. I miss someone, for example a tutor, to fall back on in case of difficulties with my studying.

LR3. I have trouble processing a large amount of subject matter.

LR4. I need guidance and and clear goals to support my studying.

LR5. It is difficult for me to determine whether I master the subject matter sufficiently.

LR6. The objectives of the course are too general for me to offer any support.
